# Factors Influencing Rumour Re-Spreading in a Public Health Crisis by the Middle-Aged and Elderly Populations

**DOI:** 10.3390/ijerph17186542

**Published:** 2020-09-08

**Authors:** Zhonggen Sun, Xin Cheng, Ruilian Zhang, Bingqing Yang

**Affiliations:** 1 School of Public Administration, Hohai University, Nanjing 211100, Jiangsu, China; sunzhonggen@hhu.edu.cn (Z.S.); 1707020105@hhu.edu.cn (X.C.); yangbingqing@hhu.edu.cn (B.Y.); 2 Sustainable Minerals Institute, University of Queensland, Brisbane 4072, Australia

**Keywords:** COVID-19, middle-aged and elderly group, rumours spreading, influencing factors, social impacts

## Abstract

Due to discrimination and media literacy, middle-aged and elderly individuals have been easily reduced to marginalized groups in the identification of rumours during a public health crisis and can easily spread rumours repeatedly, which has a negative impact on pandemic prevention and social psychology. To further clarify the factors influencing their behaviours, this study used a questionnaire to survey a sample of 556 individuals in China and used multiple linear regression and analysis of variance to explore influencing factors during the coronavirus disease 2019 (COVID-19) pandemic. We found that, first, in the COVID-19 pandemic, middle-aged and elderly adults’ willingness to re-spread rumours is positively related to their degree of believing rumours and to personal anxiety and is negatively related to their rumour-discrimination ability and to their perception of serious consequences to rumour spreading. Second, the degree of believing rumours plays an intermediary role in the willingness to re-spread rumours. It plays a partial mediating role in the path of anxiety’s influence on behaviour, suggesting that an anxious person will spread a rumour even if he or she does not have a strong belief in the rumour. Third, interpersonal communication has a greater credibility and a greater willingness to re-spread than does mass communication. This suggests the importance of increasing public knowledge expertise and of reducing public panic. This also has important implications for the future design of public health policies.

## 1. Introduction

### 1.1. Background

Middle-aged and elderly groups, defined as people over 46 years old are a very important part of communication with respect to the process of spreading rumours [1,2]. This is because, first, they account for 41.38% of the current total population in China according to the 2019 statistical yearbook [3]. Second, older people have much social experience, and middle-aged people have a higher family status in China [4]; therefore, other age groups tend to listen to both groups. Third, facing the flood of pandemic-related information, middle-aged and elderly groups who have low media literacy and relatively weak cultural qualities have low rumour-discrimination ability [5,6,7,8], which leads to being more susceptible to accepting rumours and to spreading rumours. Media literacy refers to people’s ability to choose, comprehend, question, evaluate, create, produce, and respond critically when confronted with information in various media [9]. Cultural qualities are developed through education in the humanities and arts [10], which is directly proportional to educational level, and rumour-discrimination ability refers to the ability to distinguish between right and wrong information. For example, due to the nuclear leak in Japan, there were rumours that the seawater contamination had led to salt shortage in China, which caused a salt scramble in 2011. According to an analysis by the public media, the middle-aged and elderly populations played a leading role in the spread of rumours and were also the main victims [7]. During coronavirus disease 2019 (COVID-19), some widely circulated but unconfirmed news on social media, such as that salt brine mouthwash and smoked vinegar could prevent viruses, were eventually confirmed as rumours by public media and experts. According to a research in the US, people aged 45–65 share fake news three times as much as those aged 18–29 and those over 65 share fake news seven times as much as those aged 18–29 [11]. A study shows that, in the context of the increasingly sound development of new media functions, the elderly population is enjoying short videos spread through WeChat (a chat software of Shenzhen Tencent Computer Systems Company Limited, Shenzhen, China), the authenticity of which cannot be verified. These contents were often identified as rumours [12].

Fonzo and Bordia point out that the process of spreading rumours usually goes through three stages: generation, evaluation, and re-spreading [13]. The concept of rumour re-spreading is relative to the initiation of the rumour. It emphasizes the role of the information audience in the dissemination process. The focus is on its diffusion behaviour after one hears the rumour [14]. According to the existing literature, there are many variables affecting re-spreading, and the main research covers the following four aspects: the information itself, the disseminator, the individual audience, and the social environment [7,15,16]. Allport and Postman note that the importance and ambiguity of information will contribute to the spread of rumours [17]. Chours argues that, in addition to the importance and ambiguity of information that will cause the spread of rumours, individual intelligence, the level of knowledge, and moral values will also have an impact on the propagation of rumours [18]. In addition, opinion leaders [19], communication channels [20], and trust in the rumour [21] can also influence re-spreading of the rumours. There are many academic studies on the factors influencing rumour propagation. However, there is little research on the relationship between anxiety, rumour discrimination, opinion leaders, and trust in the process of rumour re-spreading as the influencing factors [14].

In terms of subjects, the research on rumours of public health events in the existing literature mainly focuses on aspects of food safety [22] and sudden occurrence [23]. The existing research groups span across all ages, and there are fewer studies on elderly adults [24]. In addition, the existing research on the influence of rumour re-spreading of various public health events has different factors [7].

On 8 December 2019, the first case of a COVID-19 infection was found in Wuhan, Hubei Province. Subsequently, COVID-19, which is a communicable disease, has spread rapidly throughout Wuhan and other cities in China. The pandemic is rampant, and it is difficult for people to discern truth and rumours in a timely and accurate manner under conditions of panic and anxiety [25]. The rumour that “Shuanghuanglian oral liquid (a famous Chinese traditional medicine, composed of honeysuckle, scutellarin, and forsythia and an antidote with the functions of relieving fever, cough, and sore throat) can inhibit COVID-19” has caused citizens across the country to quickly gather in local pharmacies to stock up on drugs [26]. The spread of rumours related to the COVID-19 pandemic has not only exacerbated the possibility of the pandemic but also has stimulated public panic and caused chaos in social order [12].

In this article, the middle-aged and elderly groups are used as the research object to help the public understand the rumour re-spreading behaviour of this group, which addresses the gap in the literature on rumour re-spreading in middle-aged and older populations. Meanwhile, in the process of rumour re-spreading, existing studies have found that anxiety level has an influential role in rumour propagation. However, the specific role of anxiety level in rumour propagation has not been discussed. Therefore, using the brine mouthwash rumour spread in COVID-19 as an example, we found that anxiety level influences rumour re-spreading behaviour in part through the degree of belief in the rumour. These findings complement research on the factors influencing rumour re-spreading and have important implications for the future design of public health policies.

### 1.2. Hypothesis Development

This paper’s theoretical foundations are rumour propagation theory and information diffusion theory.

Rumour propagation theory was first proposed by Allport and Postman and argues that rumours are the result of a combination of event importance and event ambiguity [17]. When the environment and atmosphere are full of anxiety and uncertainty, it will increase anxiety and rumours are more likely to spread. Chours subsequently revised the theory, arguing that the spread of rumour is related to event importance plus event ambiguity and to public critical thinking of crowds [18]. Public critical thinking is also an important factor that affects the spread of rumours, consisting of an individual’s intelligence, relevant level of knowledge, and moral values. Public critical thinking represents the insight into social events that comes from people’s intelligence or other relevant factors. Differences in personalities have a very different effect on the spread of rumours, which means that different moral values will affect the possibility that people believe and spread rumours.

Information diffusion theory suggests that the spread of most information is in the shape of an “S” curve. In the process of diffusion, early adopters provide necessary help for the subsequent popularity. These early adopters convince opinion leaders in their communities, often through interpersonal communication. The opinion leaders then spread their influence to audiences in their interpersonal communication sphere to allow more people to receive the information [27,28]. Moreover, information diffusion always takes place with the help of certain social networks. In the process of diffusion of information to the society, public communication through technology is effective in providing relevant knowledge and information. However, interpersonal communication is more direct and effective in persuading people to accept new information [29].

Based on Chours’s study about critical thinking of crowds, the higher the individual’s intelligence and relevant knowledge is, the less likely the rumour is to spread; the better the moral accomplishment is, the less likely the rumour is to spread. In this paper, the ability to discern rumours is used to measure the individual’s intelligence and knowledge, and the perception of serious consequences of spreading rumours is used to measure the moral values.

Previous research has found that, in the face of rumours, the audience’s cognitive ability and emotional factors will have an impact on redistribution. In particular, anxiety is one of the most important manifestations of emotional factors [30]. In early experiments, Anthony showed that anxiety is directly proportional to the number of rumours [31], and in later empirical studies, Onook et al. also suggested that audience anxiety positively affects their willingness to re-spread [32,33,34]. Therefore, the following assumptions are made:

**H1:**  The degree of anxiety of the subjects is significantly positively related to their willingness to re-spread rumours.

The ability of rumour discrimination is to some extent an innovative concept in this paper. People’s behaviour relies on their own ability to think and judge, and a weaker ability to discern rumours limits this ability to some extent [5]. In contrast, when an individual has enough relevant knowledge, such as if a person knows the origin of the virus and has protection knowledge, then they are less likely to spread rumours. Therefore, we propose the following hypothesis:

**H2:**  Subjects’ ability to discern other rumours is significantly negatively related to their willingness to re-spread rumours.

People’s behaviour is regulated by social norms and their own values [35]. If a person of high moral cultivation knows that what he or she hears is a rumour, then he or she will understand that the consequences of spreading may have bad consequences for the followers [21], such as causing physical harm, sending the followers into panic, etc., and the perception of the severity of such consequences will prevent them from spreading the rumour. A related study also corroborated the idea that moral judgments are conducive to rumour-confrontation behaviour [36]. Thus, we propose the following hypothesis:

**H3:**  The participants’ perceptions of the serious consequences of spreading rumours are significantly negatively related to their willingness to re-spread rumours.

The news value is also known as the heat brought about by the spread of information. Opinion leaders can also increase news value by increasing the heat of the discussion [37]. Opinion leaders are influential and active people in the process of information spreading and interpersonal interaction [17]. In information dissemination, they can be nongovernment organizations, experts and scholars with a certain voice, or personal media accounts with a certain number of fans. To some extent, opinion leaders are more knowledgeable, talented, credible, and virtuous. According to the information diffusion model, we think opinion leaders can facilitate the re-spreading of rumours because they have higher levels of trustworthiness [28]. Therefore, we propose the following hypothesis:

**H4:**  Having an opinion leader or not has significant differences on participants’ willingness to re-spread rumours.

It has been shown that, when people believe rumours, they instinctively spread the topics they believe in. The more people believe a rumour, the more frequently they spread the rumour [38]. Hua [33] argued that the credibility of Internet rumours positively affects the audience’s willingness to redistribute rumours. In addition, people’s perceptions can have a driving effect on their behaviour. Trust is a person’s perception of things or opinions, which will have a propulsive effect in the course of action. In the process of rumour propagation, Liu argued that the degree of user trustworthiness plays a mediating effect in the influence of network density on the willingness to propagate rumours [22]. Thus, we propose the following hypothesis:

**H5:**  The degree to which subjects believe rumours is significantly positively related to their willingness to spread the rumours.

**H6:**  Among the above factors that significantly affect participants’ willingness to spread rumours, the degree to which they believe rumours plays a mediating role.

Social network theory can infer that mass communication facilitates the spread of rumours, but interpersonal communication makes people believe them more. The former gets information from traditional media such as television, newspapers, or new media such as the Internet, and the latter gets information from familiar family and friends. Some studies have suggested that intimate social relationships have a positive effect on the willingness to spread rumours [16,20]. This research proposes the following hypothesis:

**H7:**  The communication channels have significant differences on participants’ degree of approval of rumours and their willingness to re-spread rumours.

**Figure 1:**
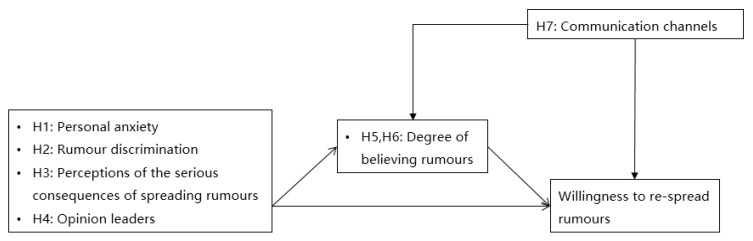
Conceptual model.

The overall conceptual model is shown in Figure 1.

## 2. Materials and Methods

### 2.1. Data Source

#### 2.1.1. Subject Selection

Most international and domestic studies use 45 years of age as the dividing line between young individuals and middle-aged individuals. Thus, this article conceptualized the middle-aged and elderly populations as those aged 46 years and older as the research sample; it was also ensured that the subjects had not heard of any information about refuting the rumours the utilized to make sure they did not know that the rumours were fake news. The article designed a questionnaire around rumours during the COVID-19 pandemic.

The questionnaire was based on the rumour that “brine mouthwash can prevent COVID-19” to test the degree to which participants in different middle-aged and elderly groups accepted the rumours and were willing to re-spread them. This rumour was selected as the case study based on the consideration that it is simple to operate and easier to implement because saltwater is readily available and thus has a herd effect. At first, the rumour was found on nearly every social media. Actually, even though brine mouthwash is a traditional and a common-sense disinfectant in Eastern culture, according to the WHO, there is no evidence that brine mouthwash can prevent COVID-19 and washing the mouth is not related to respiratory infections.

Personal anxiety was composed of three indicators: “the possibility of the virus infecting you and the people around you”, “the degree of distrust in virus prevention and control”, and “the degree of personal panic”. Rumour discrimination ability was given by seven uncertain terms related to knowledge of pandemic protection, measured by participants’ correct scores. A Likert scale was used to score the severity of the consequences of the spread of rumours and was subjectively measured by the participants. The influence of opinion leaders was measured by indicating in some questionnaires that the source of information was a certified “Dr. Returnee”, while in others, it was not clearly indicated. The level of belief and willingness to spread rumours was based on a Likert scale, with larger values indicating higher levels of belief or willingness to spread rumours.

#### 2.1.2. Questionnaire

On 18 February 2020, with the help of an Internet professional platform called “Questionnaire Star” (Ranxing Information Technology Company, Changsha, China), the questionnaire was created and geared towards middle-aged and older people over 46 years of age.

With the help of WeChat (chat software used by over 1.1 billion people in China) chat groups, we could quickly forward and recall questionnaires. Data collection of the questionnaire survey lasted for 3 days. A filtered question was set at the beginning of the questionnaire, which meant that the questionnaire was aimed at people who had not heard the brine mouthwash rumour. Only 562 respondents were eligible, and 556 questionnaires remained after eliminating duplicates, with an effective rate of 98.93%.

**Figure 2:**
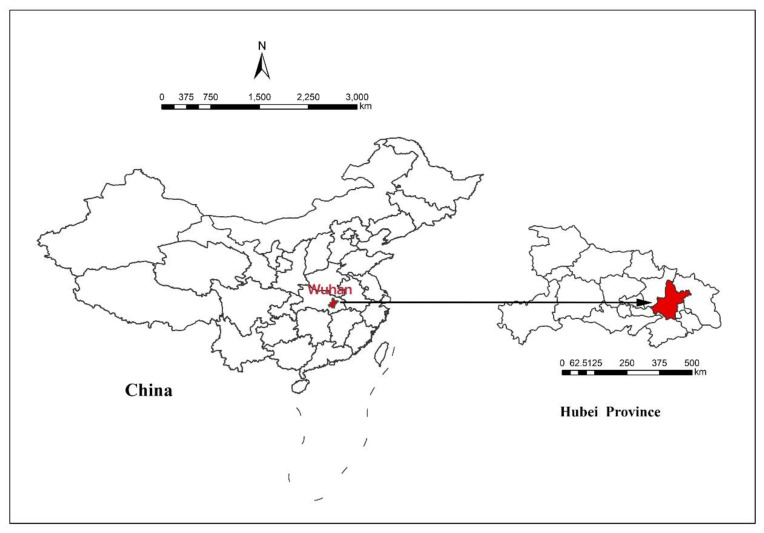
Location of Wuhan, Hubei Province.

The questionnaire was first administered in Wuhan; then, its use spread all over China, which meant that the data collected from Wuhan was supplemented by information from other provinces of China. Wuhan had the earliest outbreak of COVID-19 and the largest number of infected people. Wuhan is the capital of Hubei province, located in central China (Figure 2), with an urban resident population of 9.06 million [39].

Among the valid questionnaires recovered, 381 individuals were located in Wuhan City, Hubei Province, accounting for 68.5% of the total sample size, and the other approximately 100 questionnaires were from other provinces. According to the statistics of the pandemic situation on 21 February 2020, the cumulative number of people infected in Wuhan City, Hubei Province accounted for 79.1% of the total infected population, with fewer cases in other provinces. The proportion of the sample in Hubei Province in the valid questionnaire was close to the proportion of the population in Hubei Province with COVID-19 infection; thus, the sample is representative.

### 2.2. Methods

This study used the socioeconomic statistical analysis tool SPSS22.0 (SPSS Inc., Chicago, IL, USA) for analysis. The linear regression method was used to analyse the significant influencing factors in the model under the influence of multiple factors. The hierarchical regression method was used to detect the mediating effect of key factors, which was proposed by Baron and Kenny in 1986 [40] and is often used in the study of influence factors.

This study used personal anxiety, rumour discrimination, perception of the serious consequences of spreading rumours, and the presence of an opinion leader as independent variables. The degree of believing rumours was used as a mediator, and willingness to re-spread rumours was used as a dependent variable to build a conceptual model.

The logistics models were established as follows:

(1) $$\mathrm{Model}\;1:\;M=\alpha_{1}+\beta_{1}X_{1}+\cdots +\beta_{k}X_{i}+\varepsilon_{1}$$

(2) $$\mathrm{Model}\;2:\;Y=\alpha_{2}+\beta_{2}X_{1}+\cdots +\beta_{2k}X_{i}+\varepsilon_{2}$$

(3) $$\mathrm{Model}\;3:\;Y=\alpha_{3}+\beta_{3}X_{1}+\cdots +\beta_{3k}X_{i}+\delta M+\varepsilon_{3}$$

where Y is the dependent variable; X_i_ (i = 1,……, k) is the model’s independent variable; M is the mediator; $\;\alpha_{i},\;\beta_{i},\;\gamma_{i}$ (i = 1, 2, 3), and δ are the parameters to be estimated; and $\varepsilon_{i}\;$(i = 1, 2, 3) is the random perturbation term.

First, we conducted a regression of X on M, testing the significance of the regression coefficient $\beta_{1i}$. Second, we conducted a regression of X on Y, testing the significance of the regression coefficient $\beta_{2i}$. Third, we conducted a regression of X and M on Y, testing the significance of the regression coefficients $\beta_{3i}$ and δ. If the coefficients $\beta_{1i}$, $\beta_{2i}$, and δ are all significant, it indicates the presence of an intermediate effect. If the coefficient $\beta_{3i}$ is not significant, then the mediation effect is full; if the regression coefficient $\beta_{3i}$ is significant, but $\beta_{3i}$ < $\beta_{1i}$, then the mediation effect is partial.

To further clarify the differences in the influence of communication channels, the degree of belief in rumours and the willingness to spread rumours were set as the dependent variables, and channels were set as independent variables to build model 4. This study detected the effect of three dummy variables by means of variance analysis.

### 2.3. Ethical Approval

For this study, consent of the University Behavioral and Social Sciences Ethical Review Committee to which the researcher belongs was obtained (approval number: School of Public Administration number 20200202). All respondents were given information about the aim of the study, that the data would be treated as strictly confidential, and that all answers would be anonymous.

## 3. Results

### 3.1. Variable Descriptive Statistics

#### 3.1.1. Sample Population Attributes

As shown in Table 1, women accounted for 57.73% of the sample, men accounted for 42.27%, individuals aged 46–55 years old accounted for 57.37%, those aged 56–65 years old accounted for 24.46%, and those aged 76 years old and older accounted for 3.96%.

More than half of the participants had a bachelor’s degree or above. Considering that the outbreak occurred in the city, the questionnaire was distributed to the urban areas of Wuhan, which has 84 universities, is a major education city, and contains the top three education levels in China in 2019, with a high proportion of urban residents having an undergraduate-level education.

#### 3.1.2. Measurement of Personal Anxiety

Anxiety levels in the questionnaire were measured on a Likert scale, with 1 indicating no anxiety at all, 5 indicating being very anxious, and higher scores indicating more panic. Overall, the subjects were optimistic about the pandemic, had full confidence in virus control, and had low levels of panic (Table 2).

#### 3.1.3. Rumour Discrimination Ability

From Table 3, we find that most of the middle-aged and elderly respondents have basic protective knowledge of topics such as window ventilation and wearing masks but that only 13.13% of the participants chose all the correct answers. Of the participants, 16.01% and 8.09% believe that probiotics can improve their immunity to COVID-19 and that Shuanghuanglian oral liquid can prevent COVID-19, respectively.

#### 3.1.4. Perceived Severity of the Consequences of Rumour Spreading

A total of 74.82% of the middle-aged and elderly people respondents negatively evaluated the consequences of the spread of rumours and think that the consequences are serious or very serious. Only 2.52% of the respondents think that the spread of rumours does not matter at all (Table 4).

### 3.2. Correlation Between Major Variables

The correlation coefficients between the willingness to re-spread rumours, the degree of belief in rumours, personal anxiety, rumour discrimination, the perception of the severity of the consequences of rumour spreading, and opinion leader were all significant at the 0.05 level. Among them, there is a positive correlation between the willingness to re-spread rumours, the degree of belief in rumours, and personal anxiety. The willingness to re-spread rumours is negatively related to rumour discrimination and severity perception and positively related to opinion leaders. Among the relationships, the most obvious is the relationship between the willingness to re-spread rumours and the degree of belief in rumours, with the value reaching 0.743. Therefore, H1, H2, H3, and H4 are valid (Table 5).

### 3.3. Factors Influencing Rumour Re-Spreading

First, a linear regression was performed using personal anxiety level, rumour discrimination, perceived severity of the consequences of rumour spreading, and the presence of an opinion leader as the independent variables and the degree of believing rumours as the dependent variable to construct model 1. Model 1 illustrates that the subject’s personal anxiety (*t* = 5.346, *p* = 0.000 < 0.01), rumour discrimination (*t* = −2.292, *p* = 0.022 < 0.05), perceived severity of the consequences of rumour transmission (*t* = −2.610, *p* = 0.009 < 0.01), and the presence or absence of opinion leaders in the message (*t* = 4.632, *p* = 0.000 < 0.01) all have a significant effects on the degree of believing rumours.

Then, model 2 was developed by using willingness to re-spread as the dependent variable and by using personal anxiety level, rumour discrimination, perceived severity of the consequences of rumour spreading, and the presence of an opinion leader as the independent variables. The results of the regression analysis of model 2 show that the subjects’ personal anxiety level (*t* = 5.980, *p* = 0.000 < 0.01), rumour discrimination (*t* = −2.100, *p* = 0.036 < 0.05), perceived severity of the consequences (*t* = −2.926, *p* = 0.004 < 0.01), and the presence of opinion leaders (*t* = 3.320, *p* = 0.001 < 0.01) all significantly affect rumour re-spreading behaviour.

The results of models 1 and 2 show that personal anxiety level and the presence of opinion leaders can have a significant positive impact on the degree of belief in rumours and willingness to re-spread rumours. The ability to discern rumours and the perceived severity of the consequences of rumour spreading have a significant negative impact on the degree of belief in rumours and willingness to re-spread rumours. That is, more anxious people and those who receive information from opinion leaders are more likely to spread rumours, and the higher the rumour discernment and the clearer the perception of consequences are, the lower the likelihood that a moral person believes the rumour and spreads it.

Model 3 adds the degree of rumour belief to model 2, and this independent variable exhibits a particularly significant effect (*t* = 35.481, *p* = 0.000 < 0.01). The change in F-value shows significance (*p* < 0.01), implying that the addition of rumour level has an explanatory significance for the model. Second, the variables of rumour discrimination, the perceived severity of the consequences of rumour spreading, and the presence or absence of opinion leaders, which were originally significant, are no longer significant; third, individual anxiety level still plays a significant role but with reduced explanatory power; and fourth, the adjusted R-squared increases from 0.067 to 0.596, implying that the degree of rumour belief can generate 52.9% of the explanatory power for the willingness to re-spread rumours. The above four points indicate that the degree of belief in rumours plays a mediating role in the process of the independent variables influencing rumour re-spreading willingness (Table 6). Therefore, H5 and H6 are supported.

### 3.4. Impact of Communication Channels on the Degree of Believing Rumours and the Willingness to Re-spread Rumours

The channel of spread was used as the independent variable, and the degree of believing rumours and the willingness to re-spread rumours were analysed as the dependent variables. The kurtosis and skewness of the model were overall normally distributed. At this time, an analysis of variance was used. After the F-test, the analysis of variance showed that the type of rumour was significant for both the degree of believing rumours and the willingness to re-spread rumours (*p* < 0.01), which means that the degree of believing rumours and the willingness to re-spread rumours differ among the samples from different channels. Thus, H7 is supported (Table 7).

## 4. Discussion

Based on the findings stated above, we argue that the following aspects are critical for us to understand the impacts of COVID-19 rumour spread.

Overall, during the COVID-19 pandemic, the influencing factors of middle-aged and elderly people’s willingness to re-spread rumours includes their levels of personal anxiety, rumour discrimination, perceived severity of the consequences, the presence of an opinion leader, and the degree of believing rumours. That is, the more anxious people are, the weaker their ability to discern rumours is. In addition, the less consideration they have for others, the more trust they will have in rumours and the more willingly they will be to spread rumours [41]. This is because people’s behaviours depend on their own ability to think and judge. First of all, their personal anxiety level affects their thinking ability. When people are more anxious, it is harder for them to calmly think about the truthfulness of information [13]. Second, opinion leaders are seen as being more knowledgeable, talented, credible, and ethical than others. What they say influences the decisions of ordinary people, leading to a “spiral of silence” (If they see that the views they agree with are widely popular, they will actively participate in it and become bolder to speak out and spread) and crowd behaviour. Therefore, the presence of opinion leaders directly affects people’s trust in rumours and their willingness to re-spread them. Third, rumour discrimination allows people to improve their ability to identify rumours and to limit their re-spreading. The severity perception of the consequences of a rumour is based on moral values, which allows the recipients of the rumours to consider the perspective of others. People refrain from re-spreading rumours in order to reduce the harm they cause to others [36].

The second aspect is the degree of belief in rumours, that is, how much a person believes the rumours. The process of re-spreading rumours is a process of acceptance and retransmission, and the subjective attitude of the recipient towards the rumour influences his or her behaviour. When people believe rumours, they instinctively spread the topics they believe in [33]. With regard to the mediating role of the degree of rumour belief [22], this paper innovatively finds that, in the process of anxiety affecting their willingness to re-spread rumours, the degree of believing rumours plays a partially mediated role. However, it plays a fully mediated role in the process of rumour discrimination, in the severity perception of the consequences of rumour spreading, and in the opinion leaders’ influence on the process path of their willingness to re-spread rumours. This conclusion can be explained by two points. First, the subjects’ level of anxiety has a significant effect on their behaviour, either through the level of rumour belief or directly. The latter means that it is not replaced by the role of mediating variables and that the individual’s anxiety has an extremely important role in rumour re-spreading behaviour. Therefore, rumour re-spreading behaviour is likely to occur even if the level of rumour belief is not strong. Second, the effect of factors other than anxiety on rumour re-spreading behaviour is achieved solely by increasing the individual’s trust in rumours. In other words, the above variables cannot function if middle-aged and older adults do not believe the rumours. Suppression of rumours by mitigating panic is suggested. Community and public health departments can proactively and promptly disclose information about public health crises such as COVID-19 or can invite public health experts to give public health talks. This can increase the public’s understanding of the expertise and can reduce the public’s fears. Third, the moderating effect of communication channels is present. Studies have shown that interpersonal communication has a stronger affinity and credibility than mass communication [42]. On the one hand, the disseminator outperforms mass media in terms of network density (the closeness of the relationship between individuals) and the strength of the relationship. This increases the frequency of personal identification and communication, making the subjective level of the message more credible [22]. On the other hand, the receiving party will have their own mixed emotions and thus will be more willing to believe the other’s message. 

## 5. Conclusions

Middle-aged and older people demonstrate weakness in the areas of discrimination and media literacy and therefore easily become marginalized groups in the identification of rumours during pandemics. To understand the factors influencing middle-aged and elderly people regarding the re-spreading of rumours about COVID-19 and to clarify the influencing mechanism of different factors, we found the following after using multiple linear regression and analysis of variance. (1) In the COVID-19 pandemic, middle-aged and elderly adults’ willingness to re-spread rumours is positively related to their degree of believing rumours and personal anxiety, negatively related to their rumour discrimination ability and the perception of serious consequences, and also related to the presence of opinion leaders. The presence of opinion leaders has a direct driving effect on the degree of believing rumours and the willingness to re-spread them. (2) The degree of believing rumours plays an intermediary role in the influence on the willingness to re-spread rumours. That is, individual anxiety, rumour discrimination, perception of the severity of the consequences of rumour spreading, and the role of opinion leaders in rumour re-spreading behaviour are all achieved by increasing an individual’s trust in rumours. (3) The rumour channel is an important factor influencing the degree of believing rumours and the willingness to re-spread them. This research addresses the gap in the literature on rumour re-spreading in middle-aged and older populations and has important implications for the future design of public health policies.

The limitations of this study are as follows. First, we need more analysis of the willingness to re-spread rumours among the middle-aged; second, the sample size surveyed was limited during the rapid spread of the pandemic. It is hoped that, in subsequent relevant studies, the research on the spread of rumours among middle-aged individuals can be improved based on an adequate sample size.

## Figures and Tables

**Table 1:** Descriptive statistics of sample population attributes.

Variable	Content Composition	Percentage (%)
Gender	Male	42.27
	Female	57.73
Age	46–55 years old	57.37
	56–65 years old	24.46
	66–75 years old	14.21
	76 years old and above	3.96
Education level	Uneducated	2.70
	Primary school	7.19
	Junior high school	16.73
	High school and technical Secondary school	20.50
	Bachelor’s degree and college	45.86
	Graduate school and above	7.01

**Table 2:** Sample anxiety measurement results.

Index	Index Content	Mean
Personal anxiety	Possibility of one’s own infection and that of people in his/her environment	2.414
	Lack of confidence in virus control	1.529
	Individual panic levels	2.369
	Sum of overall panic	6.311
Cronbach’s α value	0.773	

**Table 3:** Rumour discrimination results.

Index	Option Content	Correct Rate (%)
Rumour discrimination	Need to open windows regularly to keep air flowing (right)	94.42
	Wear a mask when going out (right)	98.38
	COVID-19 can be eliminated within 30 min at 56 °C (right)	62.59
	UV disinfection lamps can kill COVID-19 (right)	41.01
	Central air conditioning will promote COVID-19 infection (right)	40.83
	Probiotics can boost immunity against COVID-19 (wrong)	83.99
	Shuanghuanglian oral liquid can prevent COVID-19 (wrong)	91.91

**Table 4:** The results of the measurement of the perceived severity of rumour spreading.

Index	Content Composition	Percentage (%)
Perceived severity of the consequences of rumour spreading	Never mind	2.52
	The consequences are not serious	6.29
	Uncertain	16.37
	The consequences are serious	50.36
	The consequences are very serious	24.46

**Table 5:** Pearson correlation coefficient table.

Variable	Average Value	Std.	Personal Anxiety	Rumour Discrimination	Perceived Severity of Rumour Spreading	The Presence of an Opinion Leader	Degree of Believing Rumours	Willingness to re-Spread Rumours
Personal anxiety	6.311	2.105	1					
Rumour discrimination	3.131	1.154	0.023	1				
Perceived severity of rumour spreading	3.879	0.934	−0.042	0.068 *	1			
The presence of an opinion leader	0.500	0.500	−0.000	−0.000	0.000	1		
Degree of believing rumours	2.310	1.070	0.158 **	−0.069 *	−0.088 **	0.135 **	1	
Willingness to re-spread rumours	1.971	1.056	0.177 **	−0.063 *	−0.097 **	0.097 **	0.743 **	1

* *p* < 0.05, ** *p* < 0.01.

**Table 6:** Hierarchical regression analysis results (*n* = 556).

Variable	Degree of Believing Rumours (Model 1)				Willingness to Re-Spread Rumours (Model 2)				Willingness to Spread Rumours (Model 3)			
	B	Std. Error	*t*	*p*	B	Std. Error	*t*	*p*	B	Std. Error	*t*	*p*
Constant	2.199 **	0.185	11.905	0.000	1.867 **	0.182	10.236	0.000	0.283 *	0.133	2.132	0.033
Anxiety level	0.080 **	0.015	5.346	0.000	0.088 **	0.015	5.980	0.000	0.031 **	0.010	3.000	0.003
Rumour discrimination	−0.062 *	0.027	−2.292	0.022	−0.056 *	0.027	−2.100	0.036	−0.011	0.018	−0.624	0.533
Perceived severity of rumour spreading	−0.088 **	0.034	−2.610	0.009	−0.097 **	0.033	−2.926	0.004	−0.034	0.023	−1.489	0.137
The presence of an opinion leader	0.290 **	0.063	4.632	0.000	0.205 **	0.062	3.320	0.001	−0.004	0.043	−0.086	0.932
Degree of believing rumours									0.721 **	0.020	35.481	0.000
R^2^	0.054				0.071				0.619			
Adjust R^2^	0.052				0.067				0.596			
F value	F(5550) = 15.914, *p* = 0.000				F (5550) = 20.992, *p* = 0.000				F (6549) = 278.129, *p* = 0.000			

* *p* < 0.05 ** *p* < 0.01.

**Table 7:** Analysis of variance results.

Model 4	Skewness	Kurtosis	Types of Rumours (Mean ± SD)		F	*p*
			Mass Communication (*n* = 556)	Interpersonal Communication (*n* = 556)		
Degree of believing rumours	−0.127	−0.978	2.17 ± 1.00	2.85 ± 1.07	229.736	0.000 **
Willingness to re-spread rumours	0.357	−1.244	1.87 ± 1.02	2.43 ± 1.21	138.396	0.000 **

** *p* < 0.01.
